# Characterization of gepotidacin activity, including *in vitro* kill kinetics, checkerboard, and PAE/SME testing against gram-positive and gram-negative bacteria

**DOI:** 10.1128/spectrum.00903-26

**Published:** 2026-08-06

**Authors:** S. J. Ryan Arends, Josh West, Nicole E. Scangarella-Oman, Mariana Castanheira, Rodrigo E. Mendes

**Affiliations:** 1 Element Iowa City (JMI Laboratories) 138461, North Liberty, Iowa, USA; 2 GSK 525885 , Collegeville, Pennsylvania, USA; Seton Hall University, South Orange, New Jersey, USA

**Keywords:** *in vitro* pharmacodynamics, urinary tract infections, gepotidacin

## Abstract

Gepotidacin is a novel, first-in-class triazaacenaphthylene antibiotic that inhibits bacterial DNA replication through a distinct binding site and unique mechanism of action that provides well-balanced inhibition of two different type II topoisomerase enzymes (DNA gyrase and topoisomerase IV) for most pathogens. Gepotidacin was recently approved by the US Food and Drug Administration (FDA) and the Medicines and Healthcare Products Regulatory Agency (MHRA) for the treatment of uncomplicated urinary tract infections (uUTI) and by the FDA for uncomplicated urogenital gonorrhea. This study investigated the *in vitro* time-kill kinetics, post-antibiotic effect (PAE), and sub-inhibitory MIC effect (PAE-SME) of gepotidacin, as well as interactions between gepotidacin and select marketed antibacterial agents for species where the published activity of gepotidacin was limited. Species included are those known to be associated with uUTI: *Citrobacter freundii* species complex, *Enterobacter cloacae* species complex, *Klebsiella aerogenes*, *Klebsiella pneumoniae*, *Proteus mirabilis*, *Providencia rettgeri*, *Enterococcus faecalis*, and *Staphylococcus saprophyticus*. Isolates were selected to represent various phenotypes, including wild type, extended-spectrum beta-lactamase positive (ESBL), and fluoroquinolone-resistant. Gepotidacin displayed concentration-dependent activity in time-kill, PAE, and PAE-SME experiments. Gepotidacin displayed bactericidal activity, often by 8 h, against 83% of isolates when tested at 4× and 10× MIC. In general, modest PAEs (>1–4 h) and extended PAE-SMEs (>4 h) were observed for gepotidacin. No antagonism was observed in broth microdilution checkerboard studies with marketed antibiotics, and the only consistent synergy observed was with gepotidacin and vancomycin against *S. saprophyticus*. These data add to the understanding of pharmacodynamics at play when treating with gepotidacin relevant uUTI species other than *Escherichia coli*.

**IMPORTANCE:**

Antibiotic resistance continues to limit treatment options for common infections such as urinary tract infections (UTIs). Gepotidacin is a recently approved antibiotic that works in a new way compared with existing drugs, allowing it to remain active against bacteria that are resistant to many commonly used treatments. In this study, we examined how effectively gepotidacin kills several bacterial species that cause UTIs, including organisms that are resistant to fluoroquinolones or produce extended-spectrum β-lactamases. Gepotidacin was shown to rapidly reduce bacterial populations at clinically relevant concentrations and continued to suppress bacterial growth even after short exposures. Importantly, gepotidacin did not interfere with the activity of other commonly used antibiotics, suggesting it can potentially be used alongside existing therapies if needed. These findings provide additional evidence supporting the effectiveness of gepotidacin against a broad range of UTI pathogens and help improve our understanding of how this new antibiotic may perform in clinical treatment.

## INTRODUCTION

Gepotidacin is a novel, first-in-class, triazaacenaphthylene antibiotic that inhibits bacterial DNA replication through a distinct binding site and unique mechanism of action that provides well-balanced inhibition of two different type II topoisomerases for most pathogens, including those resistant to standard-of-care antibiotics (1–3). Based on the results of EAGLE-2 (NCT04020341) and EAGLE-3 (NCT04187144), gepotidacin was approved in March 2025 by the US Food and Drug Administration (FDA) for the treatment of female adult and pediatric patients 12 years of age and older weighing at least 40 kg with uncomplicated urinary tract infections (uUTIs) caused by the following susceptible microorganisms: *Escherichia coli*, *Klebsiella pneumoniae*, *Citrobacter freundii* complex, *Staphylococcus saprophyticus*, and *Enterococcus faecalis* (4). Gepotidacin was further approved in August 2025 by the Medicines and Healthcare Products Regulatory Agency (MHRA) for the treatment of uncomplicated urinary tract infections (5). It has also been developed for gonorrhea; recently, a phase 3 clinical trial for the treatment of uncomplicated urogenital gonorrhea (NCT04010539 [EAGLE-1]) was completed, and FDA approval was obtained for uncomplicated urogenital gonorrhea in adult and pediatric patients 12 years of age and older (6).

Previous work was limited to investigating the *in vitro* bactericidal activity, post-antibiotic effect (PAE), and sub-inhibitory MIC effect (PAE-SME) of gepotidacin using time-kill assay methods, as well as the potential for synergy or antagonism with other classes of antibiotics via broth microdilution checkerboard assays against *E. coli*, *Neisseria gonorrhoeae*, *Staphylococcus aureus*, and *Streptococcus pneumoniae* (7, 8). This study extended that analysis to other bacterial species (additional uropathogens) for which clinical efficacy was shown (*C. freundii* species complex, *K. pneumoniae*, *E. faecalis*, and *S. saprophyticus*) (9), as well as other Enterobacterales species (*Enterobacter cloacae* species complex, *Klebsiella aerogenes*, *Proteus mirabilis*, and *Providencia rettgeri*) for which at least 90% of clinical isolates exhibit an *in vitro* MIC less than or equal to the susceptible breakpoint for gepotidacin against isolates of similar genus or organism group (5).

## MATERIALS AND METHODS

### Organisms tested

A total of 40 bacterial clinical isolates, including 5 isolates from each of the following species, were tested: *C. freundii* species complex, *E. cloacae* species complex, *K. aerogenes*, *K. pneumoniae*, *P. mirabilis*, *P. rettgeri*, *E. faecalis*, and *S. saprophyticus*. These isolates were selected from the SENTRY surveillance program and were collected from patients in 2019. Where possible, isolates were selected to include extended-spectrum β-lactamase (ESBL) and fluoroquinolone-resistant (FQ-R) phenotypes.

### Reference *in vitro* broth microdilution methods

Baseline MIC values were determined in triplicate using Clinical and Laboratory Standards Institute (CLSI) M07 (10) broth microdilution methods. QC was performed according to CLSI guidelines, and MIC values were confirmed for quality control isolates, where appropriate (11). All *in vitro* assays described in this study were conducted in cation-adjusted Mueller-Hinton broth (BD BBL, Cat# 212322).

### Time-kill kinetics

Three organisms from each species group were tested in 17 × 100 sterile test tubes with 4 mL CAMHB media containing the antibacterial agent at 0.25×, 0.5×, 1×, 2×, 4×, or 10× MIC and incubated in ambient air at 35°C. Gepotidacin time-kill cultures were sampled for CFU counts at time 0 h (T_0_), 2 h (T_2_), 4 h (T_4_), 8 h (T_8_), and 24 h (T_24_). Bactericidal activity was defined as a ≥3 log reduction in bacterial counts (log_10_ CFU/mL) (12). Aliquots from all gepotidacin time-kill cultures at the 24 h time point were plated onto 4× gepotidacin MIC agar plates to confirm changes in susceptibility to gepotidacin. Isolates recovered from the 4× gepotidacin plates were passaged twice without selection prior to screening for cross-resistance to levofloxacin, azithromycin, ceftazidime, nitrofurantoin, tetracycline, trimethoprim-sulfamethoxazole, aztreonam (gram-negative isolates only), meropenem (gram-negative isolates only), linezolid (gram-positive isolates only), and vancomycin (gram-positive isolates only). Quality control strains tested concomitantly to verify the potency of each agent and validate testing conditions included *E. coli* ATCC 25922, *P. aeruginosa* ATCC 27853, *S. aureus* ATCC 29213, and *E. faecalis* ATCC 29212.

### Post-antibiotic effect and sub-inhibitory MIC effect

Gepotidacin was tested against isolates using time-kill methods to determine PAE and PAE-SME (13–15). Similar to time-kill testing, three organisms from each species group were evaluated. Tests were carried out in 17 × 100 sterile test tubes with 10 mL CAMHB media and incubated in ambient air at 35°C. For PAE testing, organisms were exposed to gepotidacin for 1 to 2 h at 1×, 5×, and 10× the baseline broth microdilution MIC. For PAE-SME, only the 5× MIC condition was tested; 5× exposure was followed by 0.25× or 0.5× MIC in a re-exposure. Colony counts were performed at T_0_ (pre-antibacterial exposure) and T_1_ (after 1 to 2 h of antibacterial exposure). After 1 to 2 h of exposure, antibiotics were removed by centrifugation of the culture, removal of the supernatant, and resuspension in 10 mL of pre-warmed, drug-free cation-adjusted Mueller-Hinton broth for the PAE test or 10 mL of pre-warmed media containing 0.25× MIC and 0.5× MIC for the SME test. Colony counts were taken immediately after the resuspension of the organisms and then counted again at each subsequent hour for up to 9 h or until visible growth was observed. Cultures were incubated at 35°C in ambient air for the duration of the PAE and SME experiment. Untreated controls were handled in the same manner.

The duration of the PAE was calculated using the following equation: PAE was equal to T − C, where T = the time required for the CFU count in the test culture to increase 1−log_10_ above the count that was observed immediately after the drug was removed and C = the time required for the CFU count in an untreated control culture to increase 1−log_10_ above the count that was observed immediately after the same procedure that was used on the test culture for drug removal was completed.

Similarly, the duration of the PAE-SME was calculated using the following equation: SME = M − C, where M = the time required for the CFU count in the sub-inhibitory MIC test culture to increase 1−log_10_ above the count that was observed immediately after the drug was removed and the sub-inhibitory drug level was added, and C = the time required for the CFU count in an untreated control culture to increase 1−log_10_ above the count that was observed immediately after the same procedure used on the test culture for drug removal was completed.

### Synergy studies

Five organisms from each species group were evaluated. Broth microdilution panels were prepared according to methods described previously (16). Gepotidacin was tested in combination with azithromycin, ceftazidime, levofloxacin, nitrofurantoin, tetracycline, and trimethoprim-sulfamethoxazole against all isolates. Aztreonam and meropenem were only tested against gram-negative isolates, while linezolid and vancomycin were only tested against gram-positive isolates. Categorical characterization of antibacterial interaction was defined as synergy when the fractional inhibitory concentration (FIC) index was ≤0.5, indifferent at >0.5 to ≤4.0, and antagonistic at >4.0 (16). Interactions other than synergy or antagonism were referred to as indifferent or indeterminate.

Follow-up time-kill kinetic studies were carried out for any compound combinations where synergy was observed (FICs ≤ 0.5) against >50% of isolates within a species. Time-kill experiments were conducted as described above in media containing either compound or both at 0.25×, 0.5×, and 1× their respective MICs. Synergy was defined as a ≥2 log_10_ CFU/mL decrease between the combination and the most active agent alone at 24 h. Indifference and antagonism were defined at 24 h as a ±1 log_10_ to <2 log_10_ kill compared to the most active agent alone and >1 log_10_ growth compared with the less active single agent, respectively.

## RESULTS

### Baseline minimum inhibitory concentrations

Against 30 Enterobacterales isolates, the gepotidacin MIC values ranged from 0.5 to 32 µg/mL (*C. freundii* species complex, 1 to 16 µg/mL; *E. cloacae* species complex, 4 to 8 µg/mL; *K. aerogenes*, 2 to 8 µg/mL; *K. pneumoniae*, 4 to 32 µg/mL; *P. mirabilis*, 0.5 to 32 µg/mL; and *P. rettgeri*, 2 to 8 µg/mL (Table 1). For five each *E. faecalis* and *S. saprophyticus* isolates, gepotidacin MICs ranged from 0.25 to 1 and 0.06 to 0.12 µg/mL, respectively (Table 1).

**TABLE 1:** Phenotype and baseline MIC of study isolates^*a*^

Isolate	Organism	Phenotype	Test(s)	GEP	LVX	AZM	CAZ	FM	TE	SXT	ATM	MEM	LZD	VA
1091286	*C. freundii*	WT	TK, PAE, SYN	1	0.03	4	0.25	32	1	≤0.12	0.12	≤0.03	ND	ND
1103836	*C. freundii*	ESBL, FQ-R	SYN	16	2	128	>64	16	4	>16	0.25	8	ND	ND
1116313	*C. freundii*	FQ-R	TK, PAE, SYN	16	8	64	1	32	>128	>16	0.25	≤0.03	ND	ND
1130512	*C. freundii*	ESBL	TK, PAE, SYN	2	0.03	16	64	16	1	≤0.12	16	≤0.03	ND	ND
1096262	*C. koseri*	WT	SYN	1	0.03	4	0.12	32	1	≤0.12	0.06	≤0.03	ND	ND
1092279	*E. cloacae sc*	ESBL	TK, PAE, SYN	4	0.03	16	64	32	1	≤0.12	32	≤0.03	ND	ND
1092886	*E. cloacae sc*	WT	TK, PAE, SYN	4	0.03	16	1	64	1	≤0.12	1	0.06	ND	ND
1093435	*E. cloacae sc*	WT	SYN	4	0.03	8	0.5	32	2	≤0.12	1	≤0.03	ND	ND
1126581	*E. cloacae sc*	ESBL	SYN	8	0.5	16	32	64	2	>16	128	4	ND	ND
1127328	*E. cloacae sc*	FQ-R	TK, PAE, SYN	8	1	8	0.25	64	1	≤0.12	≤0.03	≤0.03	ND	ND
1089299	*K. aerogenes*	ESBL	TK, PAE, SYN	2	0.06	8	64	64	2	≤0.12	16	0.12	ND	ND
1089847	*K. aerogenes*	WT	TK, PAE, SYN	2	0.03	8	0.25	64	1	≤0.12	0.06	≤0.03	ND	ND
1092121	*K. aerogenes*	WT	SYN	2	0.03	8	0.25	64	1	≤0.12	0.12	≤0.03	ND	ND
1092937	*K. aerogenes*	FQ-R	TK, PAE, SYN	8	8	8	0.5	128	2	0.5	0.25	≤0.03	ND	ND
1106254	*K. aerogenes*	ESBL	SYN	4	0.5	8	64	64	1	0.25	128	4	ND	ND
1098581	*K. pneumoniae*	WT	TK, PAE, SYN	4	0.06	8	0.25	32	1	≤0.12	0.12	≤0.03	ND	ND
1102622	*K. pneumoniae*	WT	SYN	8	0.12	16	0.25	32	1	0.25	0.12	≤0.03	ND	ND
1107883	*K. pneumoniae*	WT	SYN	4	0.03	8	0.25	64	1	≤0.12	0.06	0.5	ND	ND
1124085	*K. pneumoniae*	FQ-R	TK, PAE, SYN	32	8	8	0.25	>128	4	2	0.12	≤0.03	ND	ND
1130299	*K. pneumoniae*	ESBL	TK, PAE, SYN	4	0.03	8	32	32	2	2	64	≤0.03	ND	ND
1091952	*P. mirabilis*	WT	TK, PAE, SYN	16	0.06	64	0.06	128	32	2	≤0.03	0.12	ND	ND
1093555	*P. mirabilis*	FQ-R	TK, SYN	8	8	64	0.06	64	16	>16	≤0.03	0.06	ND	ND
1104686	*P. mirabilis*	WT	SYN	16	0.06	64	0.06	32	32	≤0.12	≤0.03	0.06	ND	ND
1106732	*P. mirabilis*	ESBL	TK, PAE, SYN	0.5	0.5	32	2	64	32	>16	4	0.06	ND	ND
1117109	*P. mirabilis*	FQ-R, CRE	PAE, SYN	32	16	64	0.5	64	32	>16	0.5	4	ND	ND
1089529	*P. rettgeri*	WT	TK, PAE, SYN	4	0.12	128	0.06	32	32	≤0.12	≤0.03	≤0.03	ND	ND
1090192	*P. rettgeri*	ESBL, FQ-R	TK, PAE, SYN	4	16	64	>64	>128	64	>16	16	≤0.03	ND	ND
1097263	*P. rettgeri*	WT	SYN	2	0.06	32	0.06	128	32	0.25	≤0.03	0.06	ND	ND
1099742	*P. rettgeri*	ESBL, FQ-R	SYN	8	16	>128	>64	>128	32	>16	16	4	ND	ND
1118004	*P. rettgeri*	FQ-R	TK, PAE, SYN	4	8	64	0.06	>128	64	>16	≤0.03	0.06	ND	ND
1097863	*E. faecalis*	FQ-R	TK, PAE, SYN	1	32	16	>64	≤8	≤0.5	≤0.12	ND	ND	2	1
1103597	*E. faecalis*	FQ-R	SYN	0.5	32	8	>64	≤8	≤0.5	0.5	ND	ND	1	1
1103850	*E. faecalis*	WT	TK, PAE, SYN	0.25	0.5	>128	>64	≤8	32	0.5	ND	ND	1	1
1111210	*E. faecalis*	WT	TK, PAE, SYN	1	0.5	>128	>64	≤8	32	≤0.12	ND	ND	1	1
1124756	*E. faecalis*	WT	SYN	0.25	0.25	2	>64	≤8	16	≤0.12	ND	ND	2	1
1106006	*S. saprophyticus*	WT	TK, PAE, SYN	0.06	0.5	≤0.25	32	16	≤0.5	≤0.12	ND	ND	2	1
1113726	*S. saprophyticus*	WT	TK, PAE, SYN	0.06	0.5	128	>64	16	≤0.5	1	ND	ND	1	1
1115244	*S. saprophyticus*	WT	SYN	0.06	0.5	128	16	≤8	≤0.5	≤0.12	ND	ND	1	1
1125669	*S. saprophyticus*	WT	SYN	0.12	0.5	128	>64	16	≤0.5	4	ND	ND	2	1
1129086	*S. saprophyticus*	WT	TK, PAE, SYN	0.06	0.5	0.5	32	16	≤0.5	≤0.12	ND	ND	2	1
ATCC 25922	*E. coli*	WT	QC	1	0.015	4	0.25	≤8–16	1	≤0.12	0.12–0.25	0.03	ND	ND
ATCC 27853	*P. aeruginosa*	WT	QC	8	1	64	1–2	>128	16	>16	4	0.5	ND	ND
ATCC 29213	*S. aureus*	WT	QC	0.25	0.12	1	8	16	≤0.5	≤0.12	ND	ND	2	1
ATCC 29212	*E. faecalis*	WT	QC	2	1	4–8	>64	≤8	16	≤0.12	ND	ND	2	2

^*a*^ WT, wild type; ESBL, extended spectrum β-lactamase; FQ-R, fluoroquinolone resistant; CRE, carbapenem-resistant Enterobacterales; TK, time-kill; PAE, post-antibiotic and sub-inhibitory MIC effects; SYN, checkerboard synergy; QC, quality control strain; GEP, gepotidacin; LVX, levofloxacin; AZM, azithromycin; CAZ, ceftazidime; FM, nitrofurantoin; TE, tetracycline; SXT, trimethoprim-sulfamethoxazole; ATM, aztreonam; MEM, meropenem; LZD, linezolid; VA, vancomycin; ND, not determined.

### Time-kill kinetics

**Fig 1:**
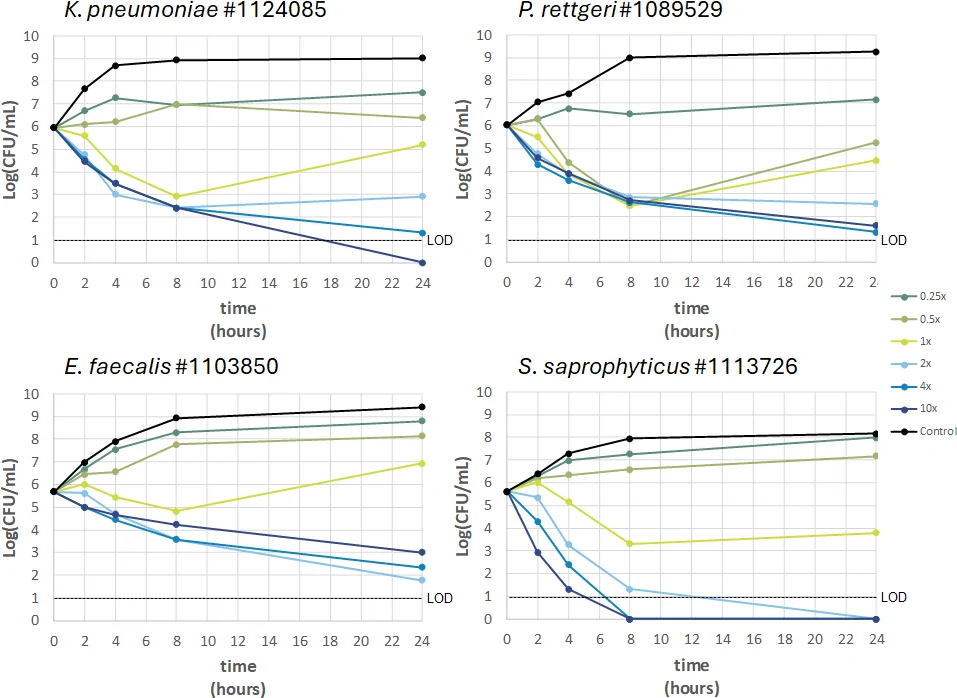
Representative time-kill curves of gepotidacin against *K. pneumoniae*, *P. rettgeri*, *E. faecalis*, and *S. saprophyticus* clinical isolates. CFUs/mL over time for each isolate were determined for various multiples of the respective gepotidacin MIC, including 0.25×, 0.5×, 1×, 2×, 4×, 10×, and the no drug control. LOD represents the limit of detection.

Against the Enterobacterales included in this study, a 3 log_10_ decrease in CFUs was maintained for 16 of 18 (89%) isolates at the 10× MIC concentration at the 24 h time point (Table 2). Representative time-kill curves for *K. pneumoniae* and *P. rettgeri* are shown in Fig. 1. Gepotidacin also demonstrated at least a 3 log_10_ decrease in bacterial CFUs against 15 of 18 (83%) isolates at the 4× MIC concentration by the 24 h time point. A 3 log_10_ decrease in CFUs was observed at lower percentages of 44% (8 of 18) and 11% (2 of 18) when tested at the 2× and 1× MIC concentrations, respectively. Most isolates showed an increase in CFUs at sub-MIC concentrations of gepotidacin.

**TABLE 2:** Log_10_ difference in CFUs observed in time-kill experiments at the 24 h time point^*a*^

Isolate no.	Organism	Key phenotype	Gepotidacin concentration (relative to MIC)					
			1/4×	1/2×	1×	2×	4×	10×
1091286	*C. freundii* sc	WT	−2.2	−1.2	−1.6	−0.8	**4.4**	**5.7**
1130512	*C. freundii* sc	ESBL	−1.6	−1.1	2.1	2.8	2.0	1.4
1116313	*C. freundii* sc	FQ-R	−1.7	−1.3	−0.4	**5.9**	**3.6**	**3.7**
1092886	*E. cloacae* sc	WT	-2	−1.8	−1.5	1.9	2.9	**3.1**
1092279	*E. cloacae* sc	ESBL	−1.7	−2.2	−1.2	−0.9	**5.7**	**5.7**
1127328	*E. cloacae* sc	FQ-R	-1	−0.1	2.1	**4.1**	**4.4**	**4.4**
1089847	*K. aerogenes*	WT	−1.2	0.2	0.2	**4.4**	**5.7**	**5.7**
1089299	*K. aerogenes*	ESBL	-1	1.2	1.5	2.9	1.8	1.1
1092937	*K. aerogenes*	FQ-R	−0.1	0.6	**4.6**	**4.3**	**4.1**	**4.6**
1098581	*K. pneumoniae*	WT	−2.1	−1.4	−2.4	−0.6	**3.9**	**5.9**
1130299	*K. pneumoniae*	ESBL	−1.7	−0.9	−0.2	0.2	**3.1**	**3.5**
1124085	*K. pneumoniae*	FQ-R	−1.6	−0.4	0.7	**3.0**	**4.6**	**5.9**
1091952	*P. mirabilis*	WT	-1	−0.2	0.1	0.8	**5.7**	**5.7**
1106732	*P. mirabilis*	ESBL	−1.1	−1.7	−0.8	0.3	**5.2**	**5.2**
1093555	*P. mirabilis*	FQ-R	−1.7	−2.6	−2.3	0.7	**3.8**	**5.1**
1089529	*P. rettgeri*	WT	−1.1	0.8	1.6	**3.5**	**4.7**	**4.4**
1090192	*P. rettgeri*	ESBL	−0.6	2.6	**6.1**	**6.1**	**6.1**	**6.1**
1118004	*P. rettgeri*	FQ-R	−0.9	−0.5	1.8	**3.5**	**3.6**	**5.7**
1103850	*E. faecalis*	WT	−3.1	−2.5	−1.2	**3.9**	**3.3**	2.7
1111210	*E. faecalis*	WT	−1.9	−1.2	1.6	**3.6**	**4.2**	**3.7**
1097863	*E. faecalis*	FQ-R	−2.1	−1.2	0.9	2.8	2.6	2.7
1106006	*S. saprophyticus*	WT	−2.6	−2.6	1.7	**5.5**	**5.5**	**5.5**
1113726	*S. saprophyticus*	WT	−2.4	−1.5	1.8	**5.6**	**5.6**	**5.6**
1129086	*S. saprophyticus*	WT	−3.0	−2.6	−1.8	0.9	**5.7**	**5.7**

^*a*^ sc, species complex; WT, wild type; ESBL, extended-spectrum β-lactamase; FQ-R, fluoroquinolone resistant. Values represent change in CFUs and were calculated by CFUs at the experiment start—CFUs at the 24 h time point. Bolded values represent a ≥3-log_10_ decrease in CFUs compared to the starting inoculum. Negative values (−) indicate growth and higher CFUs compared to the starting inoculum.

For each Enterobacterales species tested, at least one example was observed where a 3 log_10_ drop in CFUs was observed by the 4 and 8 h time points for concentrations closer to the MIC, but this drop was not maintained due to regrowth of the bacteria by the 24 h time point. Regrowth, defined as at least a 1 log_10_ drop in viable cells from the starting inoculum, followed by at least a 2 log_10_ increase in CFUs, was only seen with testing conditions near the MIC (6% of isolates at 0.25× MIC, 44% at 0.5× MIC, 56% at 1× MIC, and 33% at 2× MIC [Table S1]).

Gepotidacin also displayed bactericidal activity against the gram-positive isolates tested in this study, *E. faecalis* and *S. saprophyticus* (Fig. 1). When gepotidacin was tested against the gram-positive isolates, a 3 log_10_ decrease in CFUs was observed for 4 of 6 (67%) isolates by 24 h at the 10× MIC concentration, including all 3 *S. saprophyticus* and 1 *E. faecalis* isolate (Table 2). A 3 log_10_ drop in CFUs was observed for 5 of 6 (83%) isolates, 3 *S. saprophyticus* and 2 *E. faecalis*, at the 4× gepotidacin MIC concentration. At 2× MIC, a 3 log_10_ drop in CFUs was observed for 4 of 6 (67%) isolates, 2 *S. saprophyticus* and 2 *E. faecalis*. The largest decrease in CFUs when exposed to gepotidacin at MIC or sub-MIC concentrations (1×, 0.5×, or 0.25× MIC) was 2.3 log_10_ CFU/mL for a *S. saprophyticus* isolate at 8 h at 1× the MIC (Table S1).

### Reduced susceptibility screening and confirmatory MIC testing

Eighty-four time-kill samples were plated on agar containing 4× the baseline gepotidacin MIC to determine if reduced susceptibility to gepotidacin had developed at 24 h time-kill time points. Colonies with reduced susceptibility to gepotidacin (growth on 4× gepotidacin plates) were observed for 14 of 18 gram-negative parent isolates. The 4 parent isolates from which no isolates with reduced susceptibility were observed were 2 *K. aerogenes*, 1 *K. pneumoniae*, and 1 *P. rettgeri*. Most colonies recovered on the 4× gepotidacin plates were from time-kill testing concentrations ≤2× MIC, as there were none recovered from the 10× MIC exposure and only 4 of the 84 colonies recovered were obtained from 4× MIC exposure; 3 from a *P. mirabilis* parent and 1 from a *P. rettgeri* parent (Table S2).

Broth microdilution MIC testing showed 63 of the 82 Enterobacterales colonies were ≥8-fold less susceptible to gepotidacin compared to their respective parent strains. Of the comparator agents tested, levofloxacin was the most frequent agent to show decreased susceptibility compared to parent MIC values; however, all but one *C. freundii* complex colony, three *P. rettgeri* colonies, and eight *K. pneumoniae* colonies with decreased susceptibility to levofloxacin also showed decreased susceptibility to one or more other antibacterials (Table S2). Additional characterization of potential mechanisms responsible for these changes in susceptibility was not undertaken. It is unclear if changes were due to target-based mutations or non-specific effects like efflux or porin mutations.

Fewer colonies with reduced susceptibility to gepotidacin were recovered from the gram-positive isolates on the 4× MIC gepotidacin plates. Only two colonies, one each from *E. faecalis* (0.5× MIC) and *S. saprophyticus* (1× MIC), from all strains under all conditions were recovered on 4× MIC gepotidacin plates. Both isolates were confirmed to have gepotidacin MIC values eightfold higher than their respective parent gepotidacin MICs and all MIC values for the comparator agents against these two isolates were within fourfold of their respective parent MICs (Table S2).

### Post-antibiotic and sub-inhibitory effects

PAEs of gepotidacin were concentration-dependent for the gram-positive and gram-negative isolates included in this study (Table 3). At 1× MIC, the average gepotidacin PAE was 1.1 h, and for 12 of 24 isolates was ≤1 h. The greatest gepotidacin PAE at the 1× MIC condition was 2 h against one each *C. freundii* species complex, *E. cloacae* species complex, and *S. saprophyticus* isolate. At 5× MIC, PAEs for 5, 16, and 3 of 24 isolates were ≤1, >1 to <4, and ≥4 h, respectively. At 10× MIC, the shortest PAE was 1.5 h, with PAEs for 18 of 24 isolates at >1 to <4 h.

**TABLE 3:** Gepotidacin PAE and PAE-SME results^*a*^

Isolate no.	Organism	Key phenotype	PAE (h)			PAE-SME (h)	
			10× MIC	5× MIC	1× MIC	5× → ½× MIC	5× → ¼× MIC
1091286	*C. freundii*	WT	2.0	1.0	0.2	>6.5	3.8
1130512	*C. freundii*	ESBL	3.1	2.6	1.7	>6.4	>6.4
1116313	*C. freundii*	FQ-R	3.0	2.4	2.0	>6.9	>6.9
1092886	*E. cloacae sc*	WT	2.6	1.9	0.6	6.1	5.1
1092279	*E. cloacae sc*	ESBL	3.1	3.0	2.0	>7.2	6.4
1127328	*E. cloacae sc*	FQ-R	6.1	3.4	1.6	>7.0	>7.0
1089847	*K. aerogenes*	WT	3.0	2.9	1.3	>6.8	5.3
1089299	*K. aerogenes*	ESBL	1.7	0.7	−0.1	>6.5	3.6
1092937	*K. aerogenes*	FQ-R	4.8	4.5	1.9	>7.0	>7.0
1098581	*K. pneumoniae*	WT	2.8	2.3	0.7	>7.0	4.5
1130299	*K. pneumoniae*	ESBL	1.5	1.0	0.7	4.7	3.3
1124085	*K. pneumoniae*	FQ-R	>6.6	>6.6	1.3	>6.6	>6.6
1091952	*P. mirabilis*	WT	>6.7	3.0	0.9	>6.7	>6.7
1106732	*P. mirabilis*	ESBL	2.2	1.4	0.8	3.1	2.2
1117109	*P. mirabilis*	FQ-R, CRE	3.9	2.4	1.0	>6.8	3.7
1089529	*P. rettgeri*	WT	>6.4	0.3	0.6	>6.4	>6.4
1090192	*P. rettgeri*	ESBL	2.3	0.7	0.7	>6.4	>6.4
1118004	*P. rettgeri*	FQ-R	2.7	2.2	1.4	>6.6	>6.6
1103850	*E. faecalis*	WT	3.4	2.7	1.2	>7.0	4.4
1111210	*E. faecalis*	WT	2.0	1.2	−0.3	>5	3.8
1097863	*E. faecalis*	FQ-R	3.2	2.4	1.4	>5.9	>5.9
1106006	*S. saprophyticus*	WT	5.2	4.3	2.0	>6.1	>6.1
1113726	*S. saprophyticus*	WT	3.7	3.6	1.7	>6.1	>6.1
1129086	*S. saprophyticus*	WT	3.1	2.7	0.9	>5.7	4.1

^*a*^ sc, species complex; WT, wild type; ESBL, extended-spectrum β-lactamase; FQ-R, fluoroquinolone resistant; CRE, carbapenem-resistant Enterobacterales.

Generally, PAE-SMEs greater than 4 h were observed when the organisms that were previously exposed to 5× MIC for 1–2 h were challenged with sub-MIC concentrations (Table 3). When challenged with 0.5× MIC after exposure to 5× MIC, 21 of 24 isolates failed to increase by 1 log_10_ in CFUs, resulting in off-scale PAE values ranging from >5 to >7.2 h. The range of PAE-SME values observed at 0.5× MIC among the three isolates with on-scale results was 3.1 to 6.1 h. When challenged with 0.25× MIC after exposure to 5× MIC, 12 of 24 isolates failed to increase 1 log_10_ in CFUs, resulting in off-scale PAE values ranging from >5.9 to >7.0 h. The range of PAE-SME values observed at 0.25× MIC among the 12 isolates with on-scale results was 2.2 to 6.4 h, with an average result of 4.2 h.

### Synergy

Results of checkerboard studies with gepotidacin tested in combination with azithromycin, aztreonam, ceftazidime, levofloxacin, linezolid, meropenem, nitrofurantoin, tetracycline, trimethoprim-sulfamethoxazole, and vancomycin are summarized in Table 4. For all study isolates, combinations that were not synergistic were either indifferent or indeterminate. No antagonism was observed with any combination against any of the isolates (gram-negative or gram-positive) in this study. FIC calculations for all isolates and drug combinations can be found in Table S3.

**TABLE 4:** Number of observations of indifferences and synergies observed for gepotidacin in combination with other antibacterial agents among the five isolates from each species group tested^*a*^

Codrug	Organism group							
Interpretation	CIT	ECLC	KAE	KPN	PM	PVR	EF	SSAP
Azithromycin								
Indif./Indet.	5	5	4	5	4	5	5	5
Synergy	0	0	1	0	1	0	0	0
Ceftazidime								
Indif./Indet.	4	3	5	5	4	4	5	5
Synergy	1	2	0	0	1	1	0	0
Levofloxacin								
Indif./Indet.	5	5	5	5	4	5	5	5
Synergy	0	0	0	0	1	0	0	0
Nitrofurantoin								
Indif./Indet.	5	5	5	5	5	4	5	5
Synergy	0	0	0	0	0	1	0	0
Tetracycline								
Indif./Indet.	5	5	5	5	5	4	5	5
Synergy	0	0	0	0	0	1	0	0
TMP-SMX								
Indif./Indet.	5	5	5	3	5	5	5	5
Synergy	0	0	0	2	0	0	0	0
Aztreonam								
Indif./Indet.	4	3	5	5	5	4	ND	ND
Synergy	1	2	0	0	0	1	ND	ND
Meropenem								
Indif./Indet.	4	4	4	5	5	4	ND	ND
Synergy	1	1	1	0	0	1	ND	ND
Linezolid								
Indif./Indet.	ND	ND	ND	ND	ND	ND	5	5
Synergy	ND	ND	ND	ND	ND	ND	0	0
Vancomycin								
Indif./Indet.	ND	ND	ND	ND	ND	ND	5	1
Synergy	ND	ND	ND	ND	ND	ND	0	4

^*a*^ CIT, *Citrobacter* species; ECLC, *E. cloacae* species complex; KAE, *K. aerogenes*; KPN, *K. pneumoniae*; PM, *P. mirabilis*; PVR, *P. rettgeri*; EF, *E. faecalis*; SSAP, *S. saprophyticus*; Indif./Indet., indifference/indeterminate; TMP-SMX, trimethoprim-sulfamethoxazole; ND, not determined.

No consistent synergy was observed against the Enterobacterales tested in this study with synergy observed for only 7.5% of all combinations tested (Table 4).

**Fig 2:**
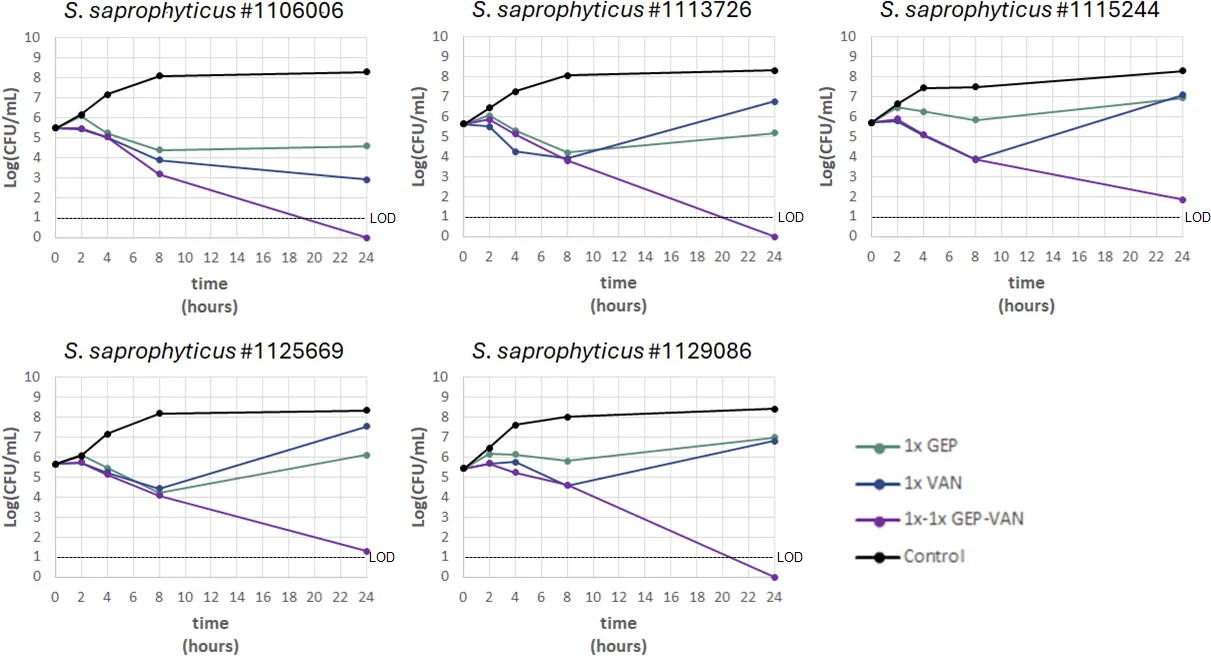
Demonstration of synergy for combination of gepotidacin and vancomycin by bactericidal time-kill curves for gepotidacin, vancomycin, and gepotidacin and vancomycin in combination at 1× the MIC against *Staphylococcus saprophyticus* isolates. LOD represents the limit of detection.

Among the gram-positive isolates, all combinations tested against *E. faecalis* resulted in indifferent or indeterminate interpretations. Against *S. saprophyticus*, gepotidacin/vancomycin was the only combination to display synergy against 4/5 isolates. The gepotidacin/vancomycin combination was further evaluated via time-kill assay against all five *S. saprophyticus* isolates. All five isolates showed ≥2 log_10_ CFU/mL decrease between the combination and the most active agent alone at 24 h at their respective 1× MIC concentrations (Fig. 2). A ≥2 log_10_ CFU/mL decrease between the combination and the most active agent alone at 24 h was not observed at the 0.25× and 0.5× MIC conditions (Table S4).

### Limitations

Though isolates were selected to represent diverse phenotypes, including ESBL-producing and/or fluoroquinolone-resistant subsets, these sample sizes limit the overall generalizability of the findings across broader contemporary clinical populations. Also, instances of colonies with reduced susceptibility to gepotidacin were recovered from several Enterobacterales isolates following exposure at gepotidacin concentrations near the MIC, molecular characterization of the underlying resistance mechanisms was not performed as part of the present study. Therefore, it remains unclear whether these changes were mediated by target-site mutations, altered efflux, permeability changes, or other mechanisms.

## DISCUSSION

The purpose of this study was to evaluate gepotidacin’s *in vitro* activity against the following organisms known to be implicated in uUTI: *C. freundii* species complex, *E. cloacae* species complex, *K. aerogenes*, *K. pneumoniae*, *P. mirabilis*, *P. rettgeri*, *E. faecalis*, and *S. saprophyticus*. Gepotidacin activity was evaluated using standard *in vitro* methods, including time-kill kinetics, PAE, PAE-SME, and broth microdilution checkerboard studies.

For all species tested, time-kill assays showed that gepotidacin was bactericidal in a dose-dependent manner, with most isolates showing a 3 log_10_ decrease in CFU mL below the starting inoculum at 4× and 10× MIC. These results are similar to those previously presented for *E. coli*, *S. aureus*, and *S. pneumoniae* (7). Similar to previous observations, isolates exposed to gepotidacin at 1× and 2× MIC often showed inhibition, followed by regrowth after 24 h. In this study, multiple colonies with reduced susceptibility to gepotidacin were recovered from most Enterobacterales parent isolates. However, only four colonies were recovered from 4× MIC exposure, with the remainder of colonies recovered from ≤2× MIC exposure. While further molecular characterization to determine the mechanism of reduced susceptibility for these isolates was not performed, a gepotidacin frequency of resistance study for these species that included characterization of resistance mechanisms has been completed (17). Recovery of gram-positive colonies with reduced susceptibility to gepotidacin was rare. Only two colonies from all conditions were recovered and confirmed to have an eightfold increase in gepotidacin MIC compared to their respective parent MIC value. While the elevated MIC values for these isolates were confirmed, molecular characterization to determine the mechanism of reduced susceptibility for these isolates was not performed.

The greater bactericidal activity and limited subsequent reduced susceptibility observed at 4× and 10× MIC align with previous characterization(s) of gepotidacin activity (18). Free-drug AUC/MIC ratio has been shown to be the PK-PD driver for gepotidacin, and amplification of resistant mutants can occur during lower exposures to gepotidacin (19). In the present study, sustained bactericidal activity was most consistently observed at 4× and 10× MIC exposures, whereas regrowth was more frequently observed at 1× and 2× MIC. These findings are clinically relevant in the context of gepotidacin treatment for uUTI, where oral administration of 1,500 mg twice daily achieves high urinary concentrations remaining well above the MICs observed for most contemporary uropathogens, including those in this study (20). In the phase 2a uUTI study supporting dose selection for the EAGLE clinical development program, trough urinary gepotidacin concentrations generally exceeded 300 µg/mL after repeated dosing (20). Relative to the MIC values of isolates tested here, these urinary exposures would correspond to sustained concentrations well above the 10× MIC conditions associated with the most rapid and durable bactericidal activity in the current study.

Gepotidacin’s PAE for the gram-positive and gram-negative bacteria tested in this study was predominantly modest (>1 to ≤4 h) at 5× MIC exposures and modest to extended (>4 h) at 10× MIC exposures. PAE-SMEs of >4 h were observed for most isolates previously exposed to 5× MIC and subsequently challenged with sub-MIC concentrations. These results are consistent with PAE/PAE-SME results previously observed for *E. coli*, *S. aureus*, and *S. pneumoniae* (7). Similar to the time-kill observations, the modest-to-extended PAE and prolonged PAE-SME observed following higher gepotidacin exposures may help support antibacterial activity during periods of declining drug concentration between oral doses. Collectively, these findings provide additional mechanistic support for the clinical efficacy observed in the EAGLE uUTI trials.

The potential for synergy and antagonism between gepotidacin and various classes of antibiotics (azithromycin, aztreonam, ceftazidime, levofloxacin, linezolid, meropenem, nitrofurantoin, tetracycline, trimethoprim-sulfamethoxazole, and vancomycin) was investigated by *in vitro* broth microdilution checkerboard assays. Interactions between gepotidacin and marketed antibiotics were mostly indifferent (∑FICmin values > 0.5 and ∑FICmax values ≤ 4) or indeterminate against all gram-positive and gram-negative bacteria tested in this study. And importantly, no instances of antagonism (∑FICmax values > 4) were observed for any of the gepotidacin/comparator combinations tested. The only example of consistent synergy (∑FICmin values ≤ 0.5) was observed between gepotidacin and vancomycin against 4/5 *S. saprophyticus* isolates. Follow-up time-kill analysis of this combination showed a ≥2 log_10_ CFU/mL decrease in the bacterial count when compared to the most active individual agent alone against all five *S. saprophyticus* isolates at 1× MIC for both compounds. It remains unclear if the synergy observed between gepotidacin and vancomycin extends to other species of Staphylococcus or other gram-positives, though no synergy was observed against *E. faecalis*. While interesting, the combination of gepotidacin and vancomycin lacks practical clinical utility for uUTI management, as uUTIs are treated outpatient with oral agents like gepotidacin, while vancomycin is administered intravenously.

Gepotidacin has been developed as an oral antibiotic for the treatment of uncomplicated urinary tract infection ([uUTI], acute cystitis) and urogenital gonorrhea. These results show gepotidacin displayed *in vitro* bactericidal activity against contemporary clinical isolates (with and without FQ-R and/or ESBL phenotypes) commonly associated with uUTI (*C. freundii* species complex, *E. cloacae* species complex, *K. aerogenes*, *K. pneumoniae*, *P. mirabilis*, *P. rettgeri*, *E. faecalis*, and *S. saprophyticus*). Modest to extended PAEs and extended PAE-SMEs for gepotidacin were also observed. Furthermore, no antagonistic interactions were seen with marketed antibacterial agents from various classes. This data adds to the understanding of pharmacodynamics at play when treating with gepotidacin for uUTI.

## Data Availability

Data available upon request.

## SUPPLEMENTAL MATERIAL

Tables S1 to S4 spectrum.00903-26-s0001.xlsx Supplemental tables describing CFU/mL determination at various time points from time-kill kinetic experiments, gepotidacin and comparator MIC values against isolates selected on 4× gepotidacin agar plates, ΣFICmin and ΣFICmax from checkerboard combinations of gepotidacin and codrugs, and CFU/mL determination at various time points from time-kill kinetic experiments.

ASM does not own the copyrights to Supplemental Material that may be linked to, or accessed through, an article. The authors have granted ASM a non-exclusive, world-wide license to publish the Supplemental Material files. Please contact the corresponding author directly for reuse.
